# The challenge of online learning for medical education during the COVID-19 pandemic

**DOI:** 10.5116/ijme.5f20.55f2

**Published:** 2020-08-21

**Authors:** John Sandars, Rakesh Patel

**Affiliations:** 1 Edge Hill University Medical School, Edge Hill University, Ormskirk, UK; 2 School of Medicine, University of Nottingham, Queen's Medical Centre, Nottingham, UK

**Keywords:** Online learning, medical education, COVID-19 pandemic

## Introduction

The global impact of the COVID-19 pandemic on education systems across the world has led to major and rapid changes in the provision of higher and medical education, with increasing delivery of the curriculum by online approaches.  A recent synthesis of the global responses by universities to the COVID-19 pandemic noted that the majority of universities were using online learning, but with differences between countries in the rapidity and extent of the shift.^1^ These differences were attributed to the available resources, which included the previous experience of using online learning and the availability of technology. However, the authors also noted that there were similar differences within countries and they highlighted that the current and urgent challenge for all universities was to ensure that the educational potential of online learning was optimised in each university.  This is an important message that is also highly relevant to all medical education providers, from basic (undergraduate) to postgraduate and continuing.  The editorial highlights the importance of iteratively designing online learning to ensure that the development, delivery and implementation of online learning are optimised to a specific local context.  In addition, the editorial discusses the importance of medical educators sharing their approaches in designing online learning.

### The importance of designing online learning

Designing effective online learning requires careful consideration of many inter-related factors. The factors include the previous experience and preferences of students in using online learning, the experience of the educators in the use of online learning, the available technology, the learning content and the curriculum, the instructional approach to provide activities that enhance learning and the  local context, such as the culture and available infrastructure resources.^2^  All of these factors are unique to a specific local context and optimisation of online learning requires a close alignment between the different factors within each context.

Several recent open-access practical articles have been produced that offer 'best practice' recommendations from experienced medical educators on how to design effective online learning, from development to delivery and implementation, including the increased challenge for low and middle-income countries.^3^^,^^4^ The main recommendation is the use of a rapid iterative design method, such as action research or educational design research, to ensure that each phase of the development, delivery and implementation process can be modified in response to the comments and evaluations from all of the stakeholders.^4^^,^^5^  These iterative design methods have rapid cycles of evaluation, with each evaluation informing the design of the next phase. This 'fine tuning' approach allows the progressive refinement of each phase to ensure that there is an effective alignment of the factors that are specific to the context.  The active participation of all stakeholders, including students, educators and administrators, is essential to ensure that a range of different perspectives can inform the design.^5^

### The importance of sharing experiences in designing online learning

There is an increasing number of opportunities for medical educators to rapidly report and share their own unique experience of developing, delivering and implementing online learning. Several of the major medical education journals, including Academic Medicine and Medical Education, now have dedicated open-access online pages for reporting medical education innovations during the COVID-19 pandemic. ^6^^,^^7^ These experiences can be very useful to medical educators in other contexts to inform their own design of online learning, but it is essential that the reports contain sufficient information, including a detailed description of how the innovation was used to overcome the barriers to the alignment of the different factors, such as the available technology.  This information is required to ensure that the expected benefit of the reported innovative approach can be achieved in another context.^8^ The use of structured templates for explicitly reporting evaluations and intervention studies can ensure that these reports contain sufficiently detailed information to be of use to other medical educators.^9^^,^^10^

## Conclusions

The main global challenge of online learning for medical education during the COVID-19 pandemic is to optimise the design to ensure that the potential of online learning can be fully realised. This challenge is likely to be resolved if there is careful attention to the design of online learning by using a rapid iterative method that involves all stakeholders.  This method provides the essential opportunity to refine the development, delivery and implementation in response to the specific local context.  It is also important that medical educators share their experiences of designing online learning but it is also essential that these reports contain sufficient information to be useful for medical educators in another context.

### Conflict of Interest

The authors declare that they have no conflict of interest.
